# Cardiometabolic Profile Segmentation in Ecuadorian University Students: A Multivariate Analysis of Lipid, Anthropometric, and Demographic Patterns

**DOI:** 10.3390/ijerph23040467

**Published:** 2026-04-07

**Authors:** Kevin Gabriel Armijo Valverde, Edgar Rolando Morales Caluña, María Victoria Padilla Samaniego, Katherine Denisse Suarez González

**Affiliations:** 1 Faculty of Health Sciences, State University of Milagro, Milagro 091050, Ecuador; 2 Research Group in Nutrition, Dietetics, Biotechnology, and Food Analysis, State University of Milagro, Milagro 091050, Ecuador

**Keywords:** university students, precision public health, disease prevention or management, disease risk prediction, lipidomic profiling

## Abstract

**Highlights:**

**Public health relevance—How does this work relate to a public health issue?**

- This study addresses the global burden of metabolic diseases by focusing on university students, a critical demographic in a transition period where early risk markers often go unnoticed.
- It overcomes the limitations of conventional univariate screenings by exploring the synergistic interaction among lipid profiles, anthropometric measures, and sociodemographic variables to better understand disease risk genesis.

**Public health significance—Why is this work of significance to public health?**

- The research identifies heterogeneous metabolic patterns using multivariate methodology (HJ-Biplot and Cluster Analysis) to successfully segment the population and reveal hidden risk patterns.
- It confirms a significantly higher cardiometabolic risk in the male population—specifically identifying a subgroup (Cluster 2) with dangerously low HDL-c levels and another (Cluster 1) with hypertriglyceridemia—highlighting a specific gender and regional disparity.

**Public health implications—What are the key implications or messages for practitioners, policy makers and/or researchers in public health?**

- Interventions must be personalized rather than generic; specifically, strategies for males should focus on intense exercise to increase HDL-c (for Cluster 2) and dietary corrections to control triglycerides (for Cluster 1).
- The findings provide a solid foundation for policy makers to design highly targeted preventive policies and screening programs within university settings to mitigate risk before chronic diseases develop.

**Abstract:**

Cardiovascular and metabolic diseases (CMDs) are the leading causes of global mortality. While university students represent a critical demographic for early intervention, conventional univariate screenings often fail to capture the synergistic interactions between lipid abnormalities and adiposity. This study aimed to identify and characterize multidimensional cardiometabolic phenotypes in Ecuadorian university students using multivariate exploratory techniques. A cross-sectional study was conducted with 365 students from the Coastal (n = 193) and Andean (n = 172) regions of Ecuador. Lipid profiles (TC, HDL-c, LDL-c, triglycerides), body composition (body fat percentage, visceral fat via bioelectrical impedance), and blood pressure were analyzed. Data were processed using HJ-Biplot analysis for dimensional reduction and a hybrid clustering approach (Hierarchical and K-means) for population segmentation. The HJ-Biplot explained 72.3% of the total variance. The first principal component (PC1, 49.2%) was associated with morphometric size (weight, height), while the second (PC2, 23.1%) was dominated by adiposity markers (body fat and visceral fat). Three distinct clusters were identified: Cluster 0 (27.1%, predominantly female) represented a low-risk profile with the highest HDL-c (57.5 mg/dL); Cluster 1 (26.6%, majority male) exhibited an intermediate-risk profile with the highest triglycerides (117.9 mg/dL); and Cluster 2 (46.3%, almost exclusively male and Andean-dominant) presented the highest risk, characterized by the lowest HDL-c levels (41 mg/dL) and older age. In conclusion, cardiometabolic risk is heterogeneously distributed across sex and geographical regions. Multivariate profiling allows for the detection of early metabolic vulnerability that remains undetected in traditional screenings. These findings support the implementation of targeted public health strategies tailored to the specific phenotypic and regional characteristics of the university population in Ecuador.

## 1. Introduction

Currently, cardiovascular and metabolic diseases (CMDs) constitute the leading causes of morbidity and mortality worldwide, imposing a substantial burden on global healthcare systems [1,2]. Within the etiology of these pathologies, lipid profiles, anthropometric factors, and sociodemographic characteristics serve as critical indicators of cardiometabolic risk, representing fundamental elements for the design of prevention and control strategies [3,4].

The identification of patterns in the joint behavior of lipid biomarkers—including Total Cholesterol (TC), high-density lipoprotein cholesterol (HDL-c), low-density lipoprotein cholesterol (LDL-c), and triglycerides—and anthropometric measures such as visceral fat and body composition—is crucial for developing effective management strategies [5]. However, most research remains limited to univariate analyses, which often fail to capture the synergistic interactions and the combined contribution of lipid abnormalities and adiposity-related indicators to the development of metabolic vulnerability.

Furthermore, sociodemographic factors such as sex, age, ethnicity, and regional context (Coastal vs. Andean regions) are consistently associated with disparities in obesity prevalence and cardiovascular risk in Latin American populations. Recent evidence highlights that ethnic background and regional residence exert a profound influence on metabolic health, driven by differences in genetic susceptibility, dietary patterns, and socioeconomic conditions [6,7,8]. Consequently, emphasizing these determinants provides a robust epidemiological framework for identifying cardiometabolic phenotypes in specific populations, such as Ecuadorian university students.

In this context, a multidimensional analytical approach is essential to generate evidence that guides targeted interventions and more effective health policies [9]. For instance, identifying subgroups that share similar lipid profiles but differ significantly in geographic distribution or sex enables a deeper understanding of how social and environmental determinants shape metabolic expression. Accordingly, this study hypothesizes that, amidst an escalating national trend of early metabolic burden, Ecuadorian university students exhibit heterogeneous cardiometabolic phenotypes. We propose that these phenotypes represent latent metabolic architectures characterized by the synergistic interaction of lipid and adiposity markers, which are more accurately captured through multivariate exploratory techniques than through traditional, clinical univariate risk stratification.

The HJ-Biplot and Cluster Analysis represent robust analytical tools to address the limitations of traditional methods [10]. The HJ-Biplot facilitates the visualization of complex relationships between individuals and variables in a reduced dimensional space, aiding the interpretation of interactions between body size and lipid variability [11]. Simultaneously, Cluster Analysis allows for the segmentation of homogeneous groups, identifying specific patterns that might remain undetected through univariate screening [12,13,14].

In Ecuador, the national burden of metabolic risk is increasing among young adults. The WHO STEPS Ecuador survey reported that 24.5% of adults present elevated total cholesterol, while overweight and obesity affect 63% of the population [1]. Moreover, data from ENSANUT 2018 documented rising trends in excess body weight beginning in late adolescence, suggesting early metabolic vulnerability in university-age populations [2]. These national trends underscore the urgency of profiling cardiometabolic phenotypes in students during this critical transition period.

This study seeks to apply multivariate exploratory techniques to characterize the relationships between lipid profiles, anthropometric measures, and demographic characteristics in university students from the Coastal and Andean regions of Ecuador. While sex-related differences in lipids are well documented, their interaction with regional and ethnic factors remains insufficiently explored in the Latin American context. Therefore, this research contributes to public health by identifying multidimensional phenotypes that support targeted screening strategies. Notably, the objective of this study was to identify and characterize these metabolic patterns through exploratory segmentation, rather than estimating clinical risk through predictive scores such as Framingham, thereby providing a foundational scientific basis for tailored prevention in vulnerable subgroups.

## 2. Materials and Methods

Study Setting and Institutional Selection

The Universidad Estatal de Milagro (UNEMI) and the Escuela Superior Politécnica de Chimborazo (ESPOCH) were strategically selected to represent two epidemiologically and socioeconomically distinct regions of Ecuador: the Coastal and the Andean (Sierra) regions. This comparative framework allows for the analysis of cardiometabolic risk patterns across populations exposed to differing environmental, dietary, and cultural conditions. Given that geographic and ethnic factors significantly influence lipid profiles and obesity prevalence in Latin America, including cohorts from both regions enhances the study’s capacity to identify region-specific metabolic phenotypes among Ecuadorian university students.

Study Design and Population

This research employed an exploratory, cross-sectional design aimed at identifying internal cardiometabolic risk patterns rather than estimating population-level prevalence. While the use of non-probabilistic convenience sampling precludes broad prevalence generalizations, it provides a robust basis for the exploratory detection of phenotypic variance within university settings.

Participants were recruited through institutional communication channels and classroom announcements.

The inclusion criteria required participants to be university students aged ≥ 18 years who were willing to participate and provided informed consent. To ensure the integrity of the baseline cardiometabolic profiles and minimize confounding factors, strict exclusion criteria were applied: individuals with a history of chronic diseases (e.g., diabetes, hypertension), those undergoing pharmacological treatments known to affect lipid metabolism, or those presenting acute inflammatory conditions at the time of the study were excluded. Additionally, to maintain the stability of the metabolic phenotypes, pregnant women were not included due to the physiological alterations in lipid and body composition profiles inherent to gestation.

The final sample consisted of 365 university students, including 193 from the Coastal region and 172 from the Andean region. Although the sampling was non-probabilistic, the sample size exceeds the recommended minimum thresholds for multivariate statistical techniques, such as HJ-Biplot and Cluster Analysis, ensuring adequate stability and analytical robustness. Specifically, the sample satisfies the commonly accepted subject-to-variable ratio (≥10:1), which supports the validity of the dimensional reduction and clustering procedures. This approach prioritizes the detection of early metabolic segmentation in a real-world academic environment over population-wide statistical inference.

Data Collection and Variables

Biochemical and Anthropometric Assessment

Fasting venous blood samples (8–12 h) were collected by trained clinical personnel following standardized biosafety protocols. Serum lipid concentrations were quantified using automated clinical chemistry analyzers. Specifically, Total Cholesterol was determined via the cholesterol oxidase-peroxidase (CHOD-PAP) enzymatic method, and Triglycerides (TG) were measured using the glycerol-3-phosphate oxidase-peroxidase (GPO-PAP) method. High-density lipoprotein cholesterol was quantified using a direct homogeneous enzymatic assay without precipitation. For participants with TG levels < 400 mg/dL, low-density lipoprotein cholesterol was calculated using the Friedewald equation (LDL-c = TC − HDL-c − TG/5); for those exceeding this threshold, values were excluded from the LDL-specific analysis to ensure calculation accuracy.

Anthropometric and body composition measurements were obtained via bioelectrical impedance analysis (BIA) using the InBody 270 equipment. Additionally, blood pressure was recorded using standardized digital sphygmomanometers following a 5 min rest protocol to ensure hemodynamic stability, minimizing potential white-coat effects or transient physiological variations.

Study Variables

The study variables were categorized as follows:

Sociodemographic: Sex, age, ethnicity, and region of residence.

Physical and Anthropometric: Weight, height, body fat percentage, visceral fat, and systolic/diastolic blood pressure.

Biochemical: TC, HDL-c, LDL-c, and triglycerides.

Although glycemic markers were excluded due to operational constraints, the lipid-anthropometric axis served as a validated surrogate for early cardiometabolic screening.

Assessment of Lifestyle and Confounders

To ensure the integrity of the identified metabolic phenotypes, direct confounding was addressed through strict exclusion criteria. While direct quantitative assessments of dietary intake and physical activity were not performed, participants with diagnosed metabolic or chronic conditions were excluded to reduce major confounding effects. Nevertheless, the researchers acknowledge that residual confounding related to unmeasured lifestyle behaviors (e.g., diet and exercise intensity) constitutes a limitation of the current study design, which is typical in exploratory cross-sectional analyses.

Multidimensional Cardiometabolic Risk Evaluation

Rather than relying on univariate clinical scores, this study adopted a profile-based approach. Risk patterns were identified through HJ-Biplot analysis to explore the joint structure of metabolic and anthropometric variables, followed by Cluster Analysis to segment participants into distinct phenotypic groups.

Body Mass Index (BMI) was intentionally not prioritized. Despite its common use, BMI’s inability to distinguish between lean mass and fat mass limits its accuracy in young adults [4]. Instead, this research emphasized direct adiposity indicators (body fat percentage and visceral fat) to provide a more sensitive detection of metabolic vulnerability and avoid BMI-related misclassification.

Data Processing and Statistical Analysis

Data processing and statistical analyses were executed using Python 3.10 within the Google Colab environment. Descriptive statistics were calculated for all variables, reporting means, standard deviations, medians, quartiles, and frequency distributions. All inferential analyses were conducted using a 95% confidence level, with statistical significance defined as *p* < 0.05. Where applicable, 95% confidence intervals (95% CI) were employed to estimate the precision of lipid, anthropometric, and demographic parameters.

To ensure analytical rigor, normality was assessed using the Shapiro–Wilk test. Accordingly, Analysis of Variance (ANOVA or Kruskal–Wallis) tests were applied to evaluate statistically significant differences between clusters, depending on the distribution of the variables. These criteria were applied uniformly across descriptive, clustering validation, and post hoc analyses to ensure the interpretability and reproducibility of the findings. Furthermore, while Body Mass Index (BMI) is widely used in epidemiological research, this study prioritized body fat percentage and visceral fat, as they better reflect metabolically active adiposity and cardiometabolic alterations, particularly in young adults [3,4].

To evaluate the individual predictive and discriminative capacity of cardiometabolic markers, Receiver Operating Characteristic (ROC) analysis was performed. The Area Under the Curve (AUC) was calculated for key continuous variables, including visceral fat level, total body fat percentage, and systolic blood pressure, utilizing the LDL-c profile as the reference standard for cardiovascular risk. This procedure enabled the quantification of the diagnostic utility of traditional univariate metrics and established a comparative baseline to justify the necessity of the multivariate analytical framework (HJ-Biplot and clustering). This approach highlights the inherent limitations of isolated clinical indicators in detecting early-stage metabolic vulnerability within a young university population.

Data Management and Outlier Treatment

A rigorous detection of multivariate outliers was conducted using Mahalanobis distance analysis. Extreme values, particularly within the lipid profiles, were examined for biological plausibility. Where necessary, Winsorization at the 95th percentile or justified exclusion was implemented to ensure a robust distribution of continuous variables, preventing distortions in the clustering results or the HJ-Biplot projection.

Data Transformation

Continuous variables were normalized via Z-score transformation (zero mean and unit standard deviation) to equalize the contribution of each measure to the multivariate analysis. Categorical variables were encoded using one-hot encoding. An initial descriptive analysis was performed to characterize the population’s central tendency and frequencies.

Multivariate Analysis and Segmentation

The HJ-Biplot technique was employed to project variables and individuals into a bidimensional space, allowing for the analysis of complex interactions among demographic, anthropometric, and biochemical measures. For population segmentation, a hybrid approach was applied: initial groups were identified through agglomerative hierarchical cluster analysis (using Gower distance and the average linkage method), followed by refinement via k-means clustering.

The optimal number of clusters was determined using a combination of the elbow method and silhouette coefficient to ensure both within-cluster cohesion and between-cluster separation.

The optimal number of clusters was determined and optimized using the Elbow Method and the Silhouette Index. Finally, the stability and differentiation of the clusters were validated using cohesion-separation indices and post hoc statistical tests (ANOVA or Kruskal–Wallis). It is important to note that this multidimensional profiling approach is exploratory; it does not constitute clinical cardiovascular risk prediction and was not validated against established scores such as Framingham or ASCVD [5]. While cluster validation was performed using the silhouette index and elbow method, sensitivity-based stability analyses were not conducted and are acknowledged as a limitation.

Ethical Considerations

The data were anonymized and the results reported in aggregated form, ensuring participant confidentiality and privacy. The study was approved by the Research Ethics Committee with Human Subjects of the Escuela Superior Politecnica de Chimborazo (specific ethical number: IO-07-CEISH-ESPOCH-2023), adhering to the Declaration of Helsinki, and all participants provided their written informed consent.

## 3. Results

Descriptive Statistics and Sample Characteristics

The study population comprised 365 university students, predominantly female (63.55%), with a mean age of 22.02 ± 2.50 years (range: 18–36 years; predominant range: 20–23 years). Geographically, 52.88% of participants originated from the Coastal region, while the remainder hailed from the Andean (Sierra) region, ensuring significant sociodemographic representation for the regional analysis. Regarding ethnicity, the Mestizo group was widely predominant (97.26%), followed by the Indigenous population (2.74%). In terms of marital status, 97.53% of the participants were single.

The statistical analysis of lipid profiles, anthropometric measures, and demographic variables revealed specific patterns within the cohort (Table 1). The mean Total Cholesterol (TC) was 181.10 mg/dL and mean Triglycerides were 108.69 mg/dL. While both values fall within clinically acceptable ranges for low-risk populations (<200 mg/dL and <150 mg/dL, respectively), the mean LDL-c (102.02 mg/dL) was situated at the upper limit of the desirable threshold (<100 mg/dL).

Furthermore, although the mean HDL-c (55.19 mg/dL) appears adequate, its distribution—ranging from 25 mg/dL to 355 mg/dL—and a 25th percentile of 45 mg/dL indicate that a substantial proportion of the students possess HDL-c levels near or below the threshold for cardiovascular protection. This variability underscores the necessity of the multivariate risk segmentation applied in this study. Anthropometrically, the mean weight and height of the participants were 63.14 kg and 1.61 m, respectively.

Participant Characteristics by Region

Participants from Universidad Estatal de Milagro (Coastal region) and Escuela Superior Politécnica de Chimborazo (Andean region) exhibited distinct demographic and physical profiles. Students from the Andean region were characterized by a different age distribution and significant variations in height and weight compared to those from the Coastal region.

Although not reaching overall statistical significance in all categories, the sex distribution varied between institutions, with a notably higher proportion of male participants in the Andean region. This demographic shift may partially account for the increased cardiometabolic vulnerability observed in specific Andean-dominant clusters. Such contrasts reinforce the premise that regional, biological, and sociocultural factors intersect to shape the cardiometabolic risk patterns identified through multivariate analysis.

Table 2 details the distribution of participants across both regions according to age, weight, height, and sex, highlighting the regional heterogeneity that underscores the necessity of a stratified analytical approach.

Continuous variables are presented as mean ± standard deviation (SD) and were compared between regions using the independent-samples t-test (specifically Welch’s *t*-test for unequal variances). Categorical variables, such as sex distribution, were compared using the Chi-square (χ^2^) test [χ^2^(1) = 0.695]. Normality was assessed using the Shapiro–Wilk test to determine the appropriate parametric or non-parametric approach. All tests were two-tailed, and statistical significance was set at *p* < 0.05.

Receiver Operating Characteristic (ROC) Analysis for Individual Risk Predictors

**Figure 1:**
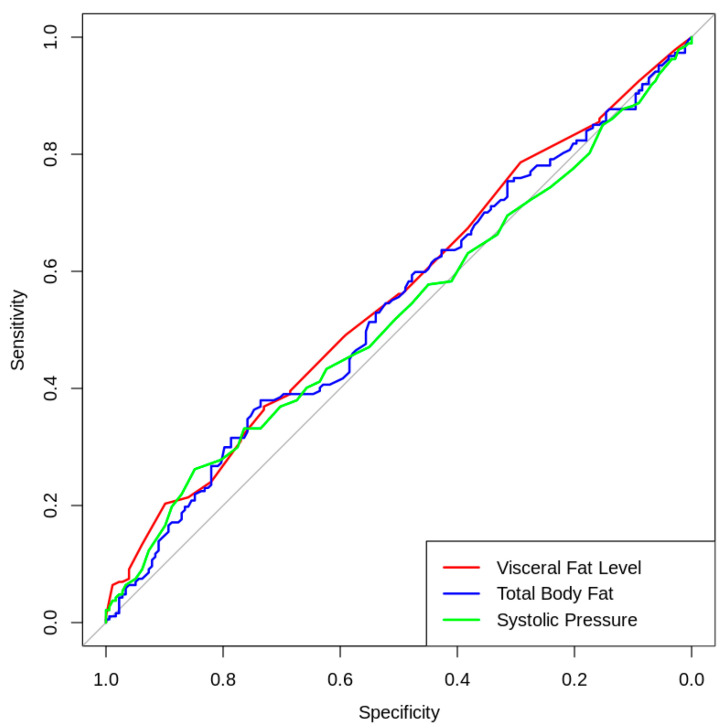
Receiver Operating Characteristic (ROC) curves evaluating the individual predictive capacity of Visceral Fat, Total Body Fat, and Systolic Blood Pressure for cardiovascular risk based on LDL-c profiles. The diagonal solid line represents the reference for a random classifier (AUC = 0.500).

To assess the predictive capacity of individual cardiometabolic markers for cardiovascular risk (defined by the LDL-c profile), a Receiver Operating Characteristic (ROC) analysis was conducted. The Area Under the Curve (AUC) was calculated for key continuous variables to evaluate their diagnostic utility. As illustrated in Figure 1, the highest AUC was observed for Visceral Fat Level (AUC = 0.558), followed by Total Body Fat (AUC = 0.542) and Systolic Blood Pressure (AUC = 0.528).

These values, being marginally above the 0.500 random-chance threshold, indicate that these traditional markers possess poor individual discriminative ability to predict specific LDL-related risk in this young university cohort. Rather than diminishing the clinical relevance of these variables, this finding underscores a critical limitation of univariate assessments in early-stage risk detection. It strongly reinforces the central premise of this study: cardiometabolic risk in young adults is a complex, non-linear, and synergistic phenomenon. Consequently, these results provide a robust statistical justification for the implementation of the multivariate segmentation (HJ-Biplot and cluster analysis) utilized in this research, which successfully captures the joint variance and latent structures of these variables.

Cluster Analysis

The cluster analysis identified three clearly differentiated cardiometabolic risk groups, each characterized by distinct lipid and anthropometric patterns. Cluster 0, composed predominantly of female students, exhibited the most favorable lipid profile, with higher HDL-c levels and lower triglycerides, indicating a less favorable lipid profile. Cluster 1, mainly composed of male students, showed elevated triglyceride concentrations, suggesting an increased metabolic risk profile potentially linked to lifestyle and dietary factors. Cluster 2, characterized by older male participants, presented the lowest HDL-c levels, representing the cluster characterized by lower HDL-c concentrations among the identified groups. These findings demonstrated meaningful heterogeneity in cardiometabolic risk patterns within the study population.

**Figure 2:**
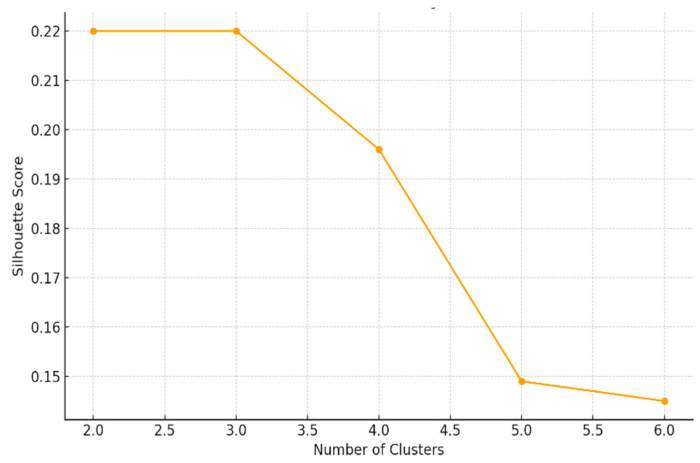
Number of optimal clusters.

The cluster analysis (optimized using the elbow method and the silhouette index, as shown in Figure 2) allowed for the identification of three differentiated groups with specific cardiometabolic risk profiles, based on the selected variables and the relationships explained by the principal components (PC1 and PC2) (see Table 3).

Cluster Characterization

Cluster 0 (N = 99, 27.12%, predominantly female, >90%): This group consists of young women (21 years on average), with lower height and weight (mean height: 1.55 m; mean weight: 55.7 k). This cluster represents the lowest risk profile, standing out for having the highest HDL-c levels (57.5 mg/dL), suggesting a healthier lipid profile. This cluster was balanced in its regional distribution (Coastal/Sierra). In the context of the principal component analysis, this group is associated with the variables dominating PC2, such as Total Body Fat (0.649) and Visceral Fat Level (0.569).

Cluster 1 (N = 97, 26.58%, majority male, >75%): This group presents intermediate characteristics in age and weight (22.9 years on average and 73.9 kg). Although this cluster showed moderate levels of TC and LDL-c, it exhibited the highest triglycerides among all clusters (117.9 mg/dL), which demands specific nutritional intervention strategies. Geographically, this cluster showed a slight prevalence of students from the Coastal region (60%). This group is strongly influenced by PC1, where variables related to body size predominate.

Cluster 2 (N = 169, 46.30%, the largest cluster, almost exclusively male, >95%): This cluster includes older men (24 years average). Despite an atypical TC value (137 mg/dL) which should be interpreted cautiously after outlier treatment, its defining characteristic is that it exhibited the lowest HDL-c (41 mg/dL), indicating the highest cardiovascular risk profile in the sample. This is the cluster with the highest prevalence of students from the Sierra region (70%). This group is also associated with the contributions of PC1, but the negative loadings of PC2 on Height (−0.340) and Total Muscle Mass (−0.278) reflect a distinct body composition, potentially indicating greater metabolic vulnerability.

HJ-Biplot Analysis

The HJ-Biplot analysis successfully reduced the multidimensional dataset into two principal components, which jointly explained 72.3% of the total variance, confirming the robustness of the dimensional reduction.

The First Principal Component (PC1) captured 49.2% of the variability and was primarily associated with morphometric characteristics such as weight (0.54), height (0.51), and age (0.34). These loadings indicate that PC1 represents a structural dimension driven by body size and age-related variability, reflecting anthropometric differentiation rather than a deterministic developmental construct.

The Second Principal Component (PC2) contributed an additional 23.1% of the variance and was dominated by adiposity markers, specifically Total Body Fat (0.649) and Visceral Fat Level (0.569). This indicates that PC2 reflects a metabolic dimension of cardiovascular risk. Notably, HDL-c exhibited a low and negative influence on both components (−0.17 on PC1 and −0.21 on PC2). In the Biplot projection, this is interpreted as an inverse or protective relationship against the majority of lipid and anthropometric risk factors.

**Figure 3:**
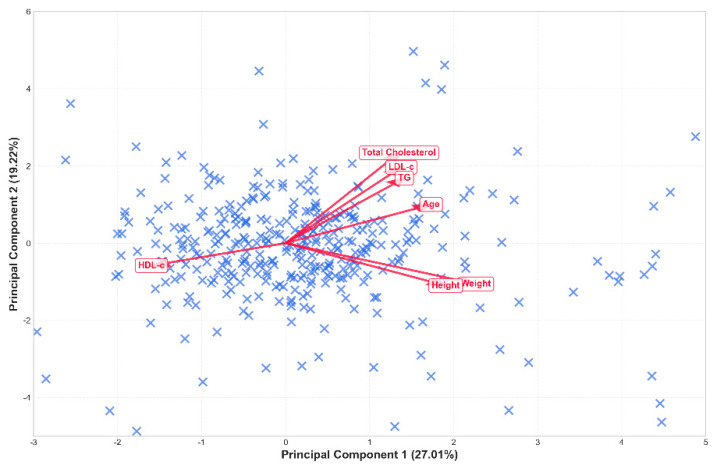
HJ-Biplot of the cardiometabolic profile. The first two axes explain 52.4% of the total inertia. Red vectors represent the variables, where their length indicates standard deviation and the cosine of the angle between them approximates the correlation between them. Blue markers (X) represent individual observations.

The vectors of the original variables in Figure 3 illustrate their contribution and direction; weight, TC, LDL-c, and triglycerides point in similar directions, confirming their direct correlation within the cardiometabolic risk framework. This spatial arrangement facilitates the understanding of the inter-variable relationships and the subsequent phenotypic segmentation of the population.

Combined Cluster and HJ-Biplot Analysis

**Figure 4:**
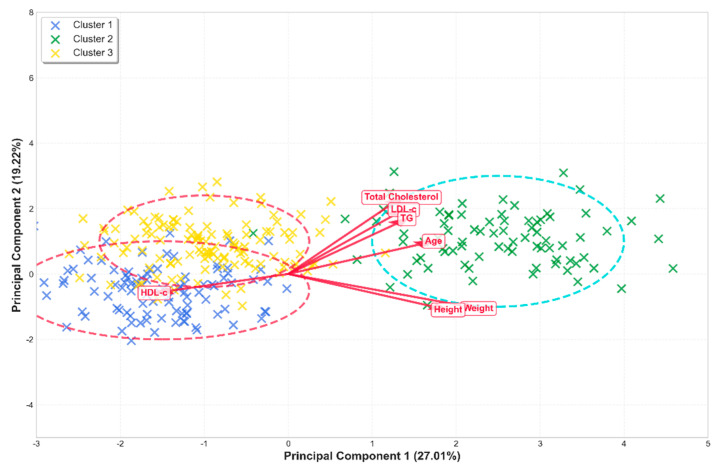
A combined cluster analysis and HJ-biplot illustrating population segmentation based on anthropometric and lipid profiles and blood pressure.

The integrated application of clustering techniques and HJ-Biplot (Figure 4) effectively segmented and visually represented the multidimensional structure of the dataset. The HJ-Biplot confirmed that the first principal component (PC1), explaining 49.2% of the variance, is primarily associated with body size and physical development. In contrast, the second principal component (PC2), contributing 23.1%, reflects the cardiovascular and adiposity-related metabolic profile.

Each cluster displayed distinct spatial patterns within the reduced dimensional space. Cluster 1 projects predominantly towards the Weight and Triglyceride vectors along PC1, aligning with its intermediate-risk profile. Conversely, Cluster 2 is situated in the region defined by the lowest projection of the HDL-c vector on PC2, visually confirming its status as the group with the highest metabolic vulnerability.

These findings provide a robust basis for population segmentation and the design of targeted public health strategies. By highlighting the synergistic relationship between body composition and lipid risk—differentiated by sex and sociodemographic region—this approach enables more precise identification of metabolic heterogeneity in young adult populations.

## 4. Discussion

This study did not incorporate established cardiovascular risk scores, such as the Framingham Risk Score or atherogenic indices, as its primary objective was exploratory segmentation rather than clinical risk prediction. Instead, a multidimensional analytical framework was applied to identify internal cardiometabolic profiles based on lipid biomarkers, anthropometric indicators, and sociodemographic characteristics. Unsupervised multivariate techniques, including HJ-Biplot and cluster analysis, focus on detecting internal data structures and relational patterns rather than estimating external population parameters. Therefore, while external generalizability is limited by the non-probabilistic sampling design, the internal validity of the identified cardiometabolic phenotypes remains robust within the target population.

Unlike traditional univariate screening approaches that rely on isolated diagnostic thresholds, the present multivariate strategy captures the covariance structure across lipid and anthropometric domains. This approach enables the detection of multidimensional phenotypes that may remain overlooked when applying single-variable criteria. By integrating body composition and lipid variability into a unified analytical framework, the HJ-Biplot–cluster approach enhances the interpretability of early metabolic heterogeneity in young adult populations. Indeed, the combined application of these techniques allowed for the segmentation and visualization of complex relationships among demographic, anthropometric, and metabolic variables. Three clearly differentiated cardiometabolic profiles were identified, consistent with previous studies demonstrating the utility of clustering for detecting metabolic heterogeneity in population-based research [15,16].

The ROC analysis further substantiated that individual anthropometric and clinical markers—specifically visceral fat level and systolic blood pressure—exhibited limited diagnostic utility, with AUC values ranging from 0.528 to 0.558. These results demonstrate that isolated univariate metrics are insufficient for early-stage risk detection in young adults, a demographic where metabolic alterations are often subclinical and subtle. This evidence directly supports the necessity of our multivariate clustering approach. Unlike traditional screenings, this method effectively uncovers latent cardiometabolic risk patterns by simultaneously accounting for the synergistic interactions between multiple biomarkers, thereby providing a more robust framework for identifying phenotypic variance in this population.

Methodologically, the first principal component (PC1) explained 49.2% of the total variance and was primarily associated with anthropometric characteristics such as weight, height, and age, reflecting a morphometric dimension. In contrast, the second principal component (PC2), which explained 23.1% of the variance, was strongly associated with lipid variables, including total cholesterol and LDL-c, representing a metabolic dimension linked to lipid-related variability. Specifically, the participants clustered in higher-risk subgroups—particularly older, male-dominant groups—demonstrated stronger projections along PC2, indicating greater cardiometabolic vulnerability characterized by dyslipidemia. These findings align with those reported by Miranda et al., who demonstrated the capacity of the HJ-Biplot to separate individuals based on anthropometric and metabolic characteristics [17], and by Park et al., who highlighted metabolic variability as a relevant risk factor for cardiometabolic diseases [18].

The visual representation provided by the HJ-Biplot was essential for interpreting the relationships among selected variables and the differentiation of risk clusters. As emphasized by Guédon et al., such visualization is valuable in public health for prioritizing prevention strategies in vulnerable subpopulations [19]. Similarly, Miranda et al. underscored the utility of HJ-Biplot techniques for evaluating the impact of nutritional and metabolic factors, reinforcing the relevance of this approach for targeted disease prevention [20].

Within this framework, a lower-risk profile, predominantly composed of younger female participants, exhibited higher HDL-c levels and lower triglyceride concentrations. In contrast, one male-dominant subgroup showed elevated triglyceride levels (117.9 mg/dL), while another presented reduced HDL-c concentrations (41 mg/dL), representing the least favorable lipid profile in the sample. These findings highlight the presence of sex-associated metabolic variability and heterogeneous lipid distribution patterns in young adults [21]. Furthermore, the overrepresentation of participants from the Andean region in the low HDL-c subgroup may reflect contextual environmental, sociocultural, or lifestyle-related factors. However, given the cross-sectional design, these findings must be interpreted as contextual associations rather than causal relationships [22,23].

From a public health perspective, these results support the relevance of early metabolic profiling in university settings. The identification of subgroup-specific patterns may inform targeted screening and health promotion strategies, particularly those focused on improving lipid profiles through lifestyle interventions. Nevertheless, these findings should be regarded as exploratory and hypothesis-generating, rather than definitive evidence for clinical guidelines [22,23]. Overall, this study demonstrates that cardiometabolic risk is heterogeneously distributed across sex, age, and regional subgroups, supporting the implementation of prevention strategies tailored to the specific metabolic and sociocultural profiles of Ecuadorian university students.

The multivariate techniques employed in this study—specifically Principal Component Analysis (PCA), HJ-Biplot, and Cluster Analysis—facilitate a comprehensive evaluation of cardiometabolic risk patterns by integrating anthropometric, lipid, and sociodemographic factors into a single analytical framework. Our findings indicate that cardiometabolic risk among Ecuadorian university students is not uniformly distributed but concentrated in specific subgroups characterized by dyslipidemia, particularly low HDL-c and elevated triglycerides in male-dominant clusters.

Segmenting the population based on multidimensional metabolic phenotypes allows for more tailored prevention strategies, supporting targeted dietary and lifestyle interventions. As suggested by Díaz and Moreno [24], such stratified approaches are essential for the effective management of chronic disease risk. This framework contributes to a more nuanced public health screening process, enabling the prioritization of resources toward high-risk subgroups (Cluster 2) where metabolic alterations are most manifest.

From a mechanistic perspective, the interplay between sociodemographic factors and regional context may influence cardiometabolic risk through the hormonal regulation of lipid metabolism, adiposity-driven inflammation, and insulin resistance. Furthermore, body composition parameters—particularly visceral fat—contribute to cardiovascular vulnerability via the release of pro-inflammatory cytokines and endothelial dysfunction, which underscores the biological plausibility of the observed cluster patterns. These mechanisms reinforce the necessity of targeted prevention, including lipid optimization and culturally tailored lifestyle interventions within university settings.

Study Limitations

Despite the robust internal validity of the multivariate patterns identified, several limitations must be acknowledged. First, the cross-sectional design precludes the establishment of causal relationships; therefore, the observed links between variables should be interpreted as associations. Second, while extreme values were controlled during the pre-processing phase, the influence of residual outliers—such as the total cholesterol levels in Cluster 2—requires cautious interpretation, a challenge inherent in multivariate modeling of biological data [25].

Additionally, this study did not incorporate established clinical tools such as the Framingham Risk Score or atherogenic indices. Our objective was exploratory segmentation to identify early metabolic vulnerability rather than longitudinal clinical prediction. Consequently, the identified clusters represent cross-sectional metabolic phenotypes rather than confirmed long-term risk categories. Furthermore, the use of a non-probabilistic sampling design and a population consisting exclusively of university students limits the generalizability of these findings. This cohort represents a relatively homogeneous group in terms of age and education, and caution is advised when extrapolating these results to the general adult population or diverse occupational groups.

Finally, certain relevant variables—including blood glucose, smoking status, dietary intake, physical activity levels, and medication use—were not available for analysis. Given the multifactorial nature of cardiometabolic risk as defined by ESC/EAS and ACC/AHA guidelines, the current clusters should be viewed as partial metabolic phenotypes. Nonetheless, they provide a valuable baseline for understanding the early heterogeneity of lipid-related risk in a critical yet understudied demographic.

## 5. Conclusions

The combined application of HJ-Biplot and Cluster Analysis provided a robust framework for identifying internally differentiated cardiometabolic phenotypes within a university population. These multivariate techniques facilitated a multidimensional characterization of lipid and anthropometric variability, transcending the limitations of isolated diagnostic thresholds.

Specifically, three distinct metabolic profiles were identified, differing significantly by sex distribution, triglyceride concentrations, and HDL-c levels. While this observed heterogeneity supports the existence of subgroup-specific metabolic patterns in young adults, these findings represent cross-sectional phenotypic segmentations rather than validated cardiovascular risk predictions.

Consequently, although these results may inform hypothesis-driven preventive strategies within academic settings, they do not support causal inferences or long-term prognostic assessments. Future research—ideally longitudinal or interventional in design—is required to incorporate dietary habits, physical activity, inflammatory markers, and validated risk scores to confirm the clinical relevance of the identified clusters.

This study demonstrates the utility of unsupervised multivariate segmentation for detecting early metabolic patterns. This approach offers a valuable methodological framework for targeted public health screening, aiding in the identification of metabolic vulnerability in young adult populations that might otherwise remain undetected through traditional univariate methods.

## Figures and Tables

**Table 1:** Descriptive statistics of the quantitative variables involved in the study.

Variable	Mean ± SD	Min	25%	50%	75%	Max
TC (mg/dL)	181.10 ± 67.48	117.00	154.00	176.00	197.00	237.00
HDL-c (mg/dL)	55.19 ± 21.14	25.00	45.00	52.00	62.00	355.00
LDL-c (mg/dL)	102.02 ± 24.78	35.00	84.00	101.00	119.00	190.00
Triglycerides (mg/dL)	108.69 ± 59.78	25.00	70.00	95.00	130.00	622.00
Weight (kg)	63.14 ± 13.99	36.20	52.50	61.90	70.20	115.60
Height (m)	1.61 ± 0.09	1.39	1.54	1.59	1.68	1.82
Age (years)	22.02 ± 2.50	18.00	20.00	22.00	23.00	36.00

TC: Total Cholesterol total, HDL-c: High-density lipoprotein cholesterol, LDL-c: Low-density lipoprotein cholesterol.

**Table 2:** Demographic distribution of participants by region.

Measure	Coastal Region	Andean Region	*p*-Value
Age (Mean ± SD)	22.36 ± 2.60	21.65 ± 2.32	0.0057
Weight (Mean ± SD)	64.62 ± 15.40	61.48 ± 12.05	0.0299
Height (Mean ± SD)	1.61 ± 0.09	1.61 ± 0.08	0.8468
Males, n (%)	66 (34.2%)	67 (39.0%)	0.4045
Females, n (%)	127 (65.8%)	105 (61.0%)	0.4045

**Table 3:** Factor loadings of variables on principal components.

Variable	PC1	PC2
Age	0.185	−0.024
Weight	0.518	0.122
Height	0.433	−0.340
Total Body Fat	0.016	0.649
Total Muscle Mass	0.487	−0.278
Visceral Fat Level	0.225	0.569
Systolic Pressure	0.405	0.100
Diastolic Pressure	0.234	0.187

## Data Availability

The data are available and are under the PROVACED research group.
